# Cross-sectional associations between early mobile device usage and problematic behaviors among school-aged children in the Hokkaido Study on Environment and Children’s Health

**DOI:** 10.1265/ehpm.22-00245

**Published:** 2023-04-12

**Authors:** Chihiro Miyashita, Keiko Yamazaki, Naomi Tamura, Atsuko Ikeda-Araki, Satoshi Suyama, Takashi Hikage, Manabu Omiya, Masahiro Mizuta, Reiko Kishi

**Affiliations:** 1 Center for Environmental and Health Sciences, Hokkaido University, Sapporo, Japan; 2 Faculty of Health Sciences, Hokkaido University, Sapporo, Japan; 3 Funded Research Division of Child and Adolescent Psychiatry, Hokkaido University Hospital, Sapporo, Japan; 4 Graduate School, Faculty of Information Science and Technology, Hokkaido University, Sapporo, Japan; 5 Information Initiative Center, Hokkaido University, Sapporo, Japan; 6 Center for Training Professors in Statistics, The Institute of Statistical Mathematics, Tokyo, Japan

**Keywords:** Hokkaido Study on Environment and Children’s Health, Mobile devices, Children, Behavioral problems

## Abstract

**Background:**

Concerns have been raised about the adverse health impacts of mobile device usage. The objective of this cross-sectional study was to examine the association between a child’s age at the first use of a mobile device and the duration of use as well as associated behavioral problems among school-aged children.

**Methods:**

This study focused on children aged 7–17 years participating in the Hokkaido Study on Environment and Children’s Health. Between October 2020 and October 2021, the participants (n = 3,021) completed a mobile device use-related questionnaire and the strengths and difficulties questionnaire (SDQ). According to the SDQ score (normal or borderline/high), the outcome variable was behavioral problems. The independent variable was child’s age at first use of a mobile device and the duration of use. Covariates included the child’s age at the time of survey, sex, sleep problems, internet addiction, health-related quality of life, and history of developmental concerns assessed at health checkups. Logistic regression analysis was performed for all children; the analysis was stratified based on the elementary, junior high, and senior high school levels.

**Results:**

According to the SDQ, children who were younger at their first use of a mobile device and used a mobile device for a longer duration represented more problematic behaviors. This association was more pronounced among elementary school children. Moreover, subscale SDQ analysis showed that hyperactivity, and peer and emotional problems among elementary school children, emotional problems among junior high school children, and conduct problems among senior high school children were related to early and long usage of mobile devices.

**Conclusions:**

Elementary school children are more sensitive to mobile device usage than older children, and early use of mobile devices may exacerbate emotional instability and oppositional behaviors in teenagers. Longitudinal follow-up studies are needed to clarify whether these problems disappear with age.

**Supplementary information:**

The online version contains supplementary material available at https://doi.org/10.1265/ehpm.22-00245.

## 1 Introduction

The usage of mobile devices, including smartphones and tablets, has rapidly increased across social classes. In 2018, the smartphone penetration rate was approximately 75% and 45% in developed and developing countries, respectively [24]. Globally, the age at which a child first uses a mobile device is constantly tipping toward earlier ages. Some parents allow their children to use mobile devices at early ages for entertainment purposes. Concerns have been raised about the negative early health impacts of regular contact with mobile devices, especially in relation to neurobehavioral developmental delays and imbalances in healthy activities in children [1, 26, 28]. The World Health Organization and some developed countries, including the United States of America, Canada, and Australia, have recommended that parents should avoid giving screen-based devices to infants younger than 18 months and restrict screen time to <1 hour daily for preschool children aged 2–5 years [2, 3, 23, 30, 31].

While these recommendations are based on previous studies that mainly targeted traditional devices such as the television, there is limited scientific evidence regarding the associations between the early use of contemporary mobile devices and development in children. Unstructured play with the hands and body and practical social communication are important for the development of the central nervous system in early infancy [2, 3]. For establishing healthy behaviors, daytime activities, nighttime sleep, and routine mealtimes are essential. Early mobile device usage can interfere with comprehensive neurodevelopment and engagement in healthy activities, both of which foster language and cognitive development and social skills in infancy. According to a Korean study, children aged 1–3 years who spent longer times with touch screens displayed increased emotional problems and depression and anxiety symptoms [15]. Another study reported that the regular use of screen-based media among infants at 4 months was associated with poor performance on self-regulation tests but not cognitive flexibility or working memory tests at assessed at 14 months [17].

In Japan, the internet penetration rate among school-aged children was 93.2% in 2019. The penetration rate rapidly increased from 12.5% to 49.5%, for smartphones and from 15.3% to 41.0%, for tablets, among elementary school students, between 2014 and 2019. More than 50% of children have contact with the internet in their early life, with 4.7% of those aged 0 years and 50.2% of those aged 3 years using the internet [18]. A Japanese study suggested that regular and frequent use of mobile devices among first grade elementary school children was associated with increased emotional and behavioral problems [9]. However, this study did not assess school children aged >7 years. The association between mobile device usage in early life and developmental effects was not evaluated. Therefore, the current study aimed to assess the association between a child’s age at first use of a mobile device and the duration of use and the behavioral problems among elementary, junior high, and senior high school students.

## 2 Methods

### 2.1 Subjects

**Fig. 1:**
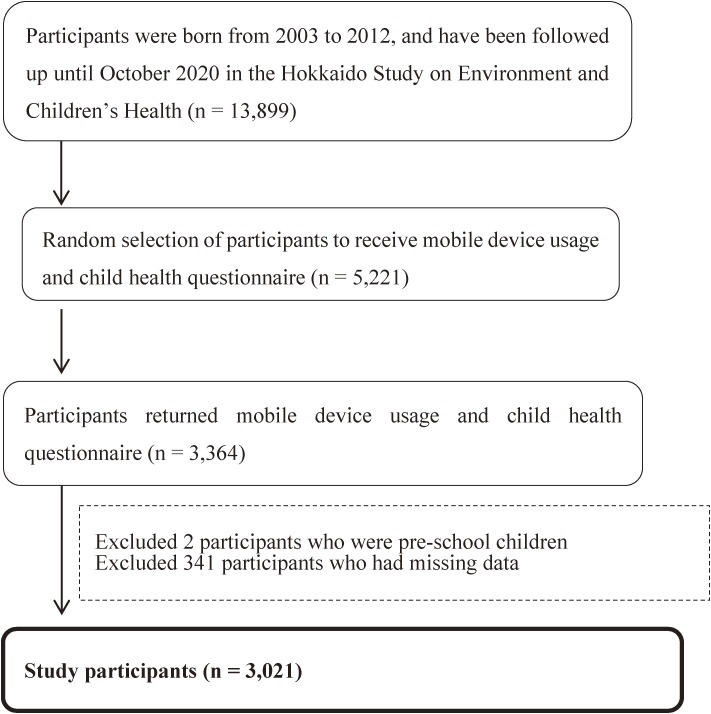
Flow chart of study participants

This study focused on children aged 7–17 years between 2003 and 2012; the children were followed up until October 2020 in a prospective birth cohort study of the Hokkaido Study on Environment and Children’s Health [11–13]. We mailed the strengths and difficulties questionnaire (SDQ) and a questionnaire regarding mobile device use and lifestyle to 5,221 parent–child pairs between October 2020 and January 2021 based on a random selection. A total of 3,364 responses were received by October 2021 (response rate = 64.4%). From the questionnaire, we assessed the associations between exposure to mobile devices (the child’s age at first use of a mobile device and duration of use), the outcomes (child behavioral problems as determined by the SDQ), and potential covariates, including the child’s sleep problems, internet addiction, health-related quality of life, and history of developmental concerns assessed at health checkups (Fig. 1). The children’s age at survey were recorded based on the response date on the questionnaire. The child’s date of birth and sex were obtained from medical records. Additional information, including the parents’ age and educational and household incomes, were obtained from the baseline questionnaire during maternal pregnancy [11, 13]. Of the 3,364 questionnaires returned, we excluded two pre-school children and 341 participants with missing response data. A total of 3,021 children, including 1,433 elementary school, 1,121 junior high school, and 467 senior high school children, were finally included in the study (Table 1 and Fig. 1).

**Table 1:** Characteristics of participants (children and their parents).

	**Categories**	**All children**		**School type**						**p**

				**Elementary school**		**Junior high school**		**Senior high school**		
			
		**Number ** **(%)**	**Mean ± SD or** **Median (IQR)**	**Number ** **(%)**	**Mean ± SD or** **Median (IQR)**	**Number ** **(%)**	**Mean ± SD or** **Median (IQR)**	**Number ** **(%)**	**Mean ± SD or** **Median (IQR)**	
Child										
Age at survey		3021	12.4 ± 2.4	1433	10.2 ± 1.2	1121	13.7 ± 0.9	467	15.8 ± 0.5	<0.001

Sex	Boy	1499 (49.6)		721 (50.3)		550 (49.1)		228 (48.8)		0.766
	Girl	1522 (50.4)		712 (49.7)		571 (50.9)		239 (51.2)		

Siblings	No	401 (16.0)		192 (17.2)		151 (15.4)		58 (14.4)		0.342
	Yes	2100 (84.0)		926 (82.8)		830 (84.6)		344 (85.6)		

History of developmental concerns	No	2726 (90.2)		1271 (88.7)		1028 (91.7)		427 (91.4)		0.025
	Yes	295 (9.8)		162 (11.3)		93 (8.3)		40 (8.6)		

Personal mobile device use and restriction										
Having personal mobile devices	No	564 (18.7)		434 (30.3)		129 (11.5)		1 (0.2)		<0.001
	Yes	2457 (81.3)		999 (69.7)		992 (88.5)		466 (99.8)		

Restricted use of mobile devices on weekdays	No	1310 (43.4)		389 (27.2)		540 (48.2)		381 (81.6)		<0.001
	Yes	1709 (56.6)		1042 (72.8)		581 (51.8)		86 (18.4)		

Restricted use of mobile devices on holidays	No	1456 (48.2)		472 (32.9)		589 (52.5)		395 (84.6)		<0.001
	Yes	1565 (51.8)		961 (67.1)		532 (47.5)		72 (15.4)		
Child health quality										
Health-related quality of life		3021	43.0 (38.0, 47.0)	1433	44.0 (40.0, 48.0)	1121	43.0 (37.0, 47.0)	467	41.0 (35.0, 46.0)	<0.001
Sleep problems		3021	24.0 (22.0, 26.0)	1433	24.0 (22.0, 26.0)	1121	23.0 (22.0, 26.0)	467	23.0 (21.0, 25.0)	<0.001
Internet addiction		3021	21.0 (16.0, 26.0)	1433	19.0 (15.0, 24.0)	1121	21.0 (17.0, 26.0)	467	23.0 (19.0, 27.0)	<0.001

Mother										
Age at time of survey		3021	44.1 ± 5.1	1433	42.1 ± 4.9	1121	45.3 ± 4.6	467	47.2 ± 4.2	<0.001

Educational level	≤9	80 (2.6)		37 (2.6)		32 (2.9)		11 (2.4)		
	10–12	1152 (38.1)		553 (38.6)		421 (37.6)		178 (38.1)		0.478
	13–15	1376 (45.5)		630 (44.0)		522 (46.6)		224 (48.0)		
	>16	413 (13.7)		213 (14.9)		146 (13.0)		54 (11.6)		
Father										
Age at time of survey		2984	45.7 ± 5.9	1419	43.7 ± 5.7	1103	46.9 ± 5.4	462	48.9 ± 5.2	<0.001

Educational level	≤9	167 (5.6)		82 (5.8)		56 (5.1)		29 (6.3)		0.637
	10–12	1099 (36.8)		508 (35.6)		409 (37.1)		182 (39.6)		
	13–15	785 (26.3)		385 (27.0)		292 (26.5)		108 (23.5)		
	>16	938 (31.4)		451 (31.6)		346 (31.4)		141 (30.7)		

Annual household income (million Japanese Yen)	<3.0	510 (19.0)		252 (19.5)		194 (19.6)		64 (15.7)		0.481
	3.0–4.9	1230 (45.8)		594 (46.0)		446 (45.1)		190 (46.7)		
	5.0–7.9	722 (26.9)		347 (26.9)		263 (26.6)		112 (27.5)		
	≥8	226 (8.4)		98 (7.6)		87 (8.8)		41 (10.1)		

SD, standard deviation. SDQ, Strength and Difficulties Questionnaire. IQR, interquartile range is the 75th and 25th percentile

p value by one-way ANOVA, χ2 test, and Kruskal-Wallis test, which were used to compare data among elementary, junior high, and senior high school children.

### 2.2 Ethics statement

The institutional ethics board for epidemiological studies at Hokkaido University Graduate School of Medicine and Hokkaido University Center for Environmental and Health Sciences approved the study protocol (approval number 19 - 118). Informed consent was obtained from all study participants before enrollment.

### 2.3 Exposure assessment

We used 4-type exposure factors to monitor child mobile device usage (Table 2); the first exposure factor was the child’s age at the first use of a mobile device, according to parents’ answer to the following question: “At what age did your child first use a mobile device? For example, you showed movies to your child on mobile devices, or your child used a mobile device by themselves?” The second exposure factor was the duration of mobile device usage, meaning usable years, calculated based on the child’s age at the first use of a mobile device and that at the time of survey. The third exposure factor was the child’s age at which they were given personal mobile devices, according to answers by parents to the following question: “At what age did your child first receive a personal mobile device such as a cell phone or tablet?” The fourth exposure factor was the duration of personal mobile device usage, indicating that holding years were calculated using the age at which a child had a personal mobile device and that at the time of survey.

**Table 2:** Child’s age at first use of a mobile device and the duration of use.

	**All children**	**School type**			**p**

		**Elementary ** **school children**	**Junior high ** **school children**	**Senior high ** **school children**	
	
	**Median (IQR)**	**Median (IQR)**	**Median (IQR)**	**Median (IQR)**	
Age at first use of a mobile device	7.0 (5.0, 10.0)	6.0 (4.0, 8.0)	9.0 (6.0, 11.0)	10.0 (7.0, 12.0)	<0.001
Duration of mobile device usage	5.0 (3.0, 7.0)	4.0 (2.0, 6.0)	5.0 (3.0, 8.0)	6.0 (3.0, 9.0)	<0.001
Age at first having a personal mobile device	12.0 (8.0, 13.0)	8.0 (7.0, 10.0)	12.0 (11.0, 13.0)	14.0 (12.0, 15.0)	<0.001
Duration for having a personal mobile device	2.0 (1.0, 3.0)	2.0 (1.0, 3.0)	2.0 (1.0, 3.0)	2.0 (1.0, 4.0)	0.49

IQR, interquartile range. p value by Kruskal-Wallis test, which were used to compare median among elementary, junior high, and senior high school children.

### 2.4 Assessment outcome

We used the SDQ of the common methods for assessing behavioral and mental health problems among children and adolescents aged 4–17 in questionnaires, which were completed by the children’s parents or teachers [7]. The SDQ consists of 25 items, each rated as being not true (0), somewhat true (1), or certainly true (2). The items are divided into five subscales covering conduct problems, hyperactivity, emotional symptoms, peer problems, and prosocial behavior [7]. Summing up the scores on the four subscales, i.e., excluding prosocial behavior, gives the SDQ total difficulties score (TDS), which can range from 0 to 40. The TDS from the Japanese version of the SDQ has a cut-off score of 12/13 for normal/borderline and 15/16 for borderline/high [16]. In this study, according to the parents’ answers, the children were divided into two groups—normal or borderline/high—based on the TDS and five subscales. The cut-off score for the five subscales of conduct problems, hyperactivity, emotional symptoms, peer problems, and prosocial behavior were 3/4, 5/6, 3/4, 3/4, and 6/5 for the normal and borderline/high groups, respectively [8].

### 2.5 Covariates

Based on previous studies, we used several covariates, including the children’s age at the time of survey, sex, and history of developmental concerns at health checkups, health-related quality of life, sleep problems, internet addiction, school type, and interaction between a child’s mobile device usage and school type, all of which had potential confounding effects on a child’s mobile device usage and child behavioral problems based on the associations noted in this study (Table 4 and Supplemental Table 2 and 3) [9, 16, 19]. In fact, we assessed the generic health-related quality of life for children using the KIDSCREEN-10 questionnaire [21]; this questionnaire consists of 10 items, including the physical, psychological, and social dimensions of wellbeing [25]. We also assessed the tendency toward internet dependence using a modified Internet Addiction Test, which comprised 11 items [29, 32]. These questionnaires were completed by the children. We assessed sleep problems in the children based on 19 items from the short version of the sleep questionnaire for children [22], which was answered by their parents. Moreover, we used covariates to assess the interactions between a child’s mobile device usage and school type. The children included in this cross-sectional study had a wide birth-year period of 10 years, and their mobile device penetration rate had rapidly changed in the meantime [18]. The children’s behavioral problems not only changed as they developed but were also related to schooling from elementary to high school. We conducted school-specific analyses because we believed that the school type affected both the exposure and outcomes. Meanwhile, we did not use covariates of parental household income, educational levels, and the presence of siblings as these factors were not associated with a child’s mobile device usage in this study (Table 4). This indicates that the association between mobile device usage and social class could be weakening.

### 2.6 Statistical analysis

Simple associations between the parents’ and children’s characteristics and the child’s age at first use of a mobile device and duration of use as well as the children’s behavioral problems were assessed using one-way ANOVA, the χ^2^ test, and the Kruskal–Wallis test. A logistic regression analysis was performed for all children, stratified by elementary, junior high, and senior high school; the outcome (TDS and sub-analyses of child behavioral problems by SDQ) was considered the dependent variable, whereas exposure (child’s age at first use of a mobile device and the duration of use) was considered the independent variable. The covariates included child age at time of survey, sex, sleep problems, internet addiction, health-related quality of life, history of developmental concerns assessed at health checkups, and school type. The logistic regression analysis among children stratified by school type was adjusted for the same variables, except for school type. p < 0.05 was considered statistically significant. All statistical analyses were performed using SPSS software for Windows (version 21.0J; IBM, Armonk, NY, USA).

## 3 Results

A total of 3,021 children, including 1,433 elementary school, 1,121 junior high school, and 467 senior high school children, were involved in this study. The children’s and parents’ characteristics and the differences among school types are shown in Table 1. The children’s and parents’ ages at the time of survey, the child’s internet addiction score, and the rate of having personal mobile devices increased as the children progressed from elementary to senior high school. The rate of restricted mobile device usage on weekdays and holidays and the health-related quality of life scores decreased as the children progressed from elementary to senior high school. Moreover, the history of developmental concerns assessed at health checkups and sleep problem scores differed among elementary, junior high, and senior high school children (Table 1).

The median age at first use of a mobile device and the duration of use was 7.0 and 5.0 years overall, 6.0 and 4.0 among elementary school children, 9.0 and 5.0 among junior high school children, and 10.0 and 6.0 among senior high school children, respectively (Table 2). The distribution of child age at first use of a mobile device is shown in Supplemental Table 1. The number of borderline/high (cases) according to TDS was 435 (14.4%) overall, 228 (15.9%) among elementary school children, 149 (13.3%) among junior high school children, and 58 (12.4%) among senior high school children (Table 3). The five subscales of childhood behavioral problems according to SDQ are shown in Table 3. The associations between the age at which a child first used a mobile device and basic participant information are shown in Table 4, both unstratified and stratified by school type. Among all children, we observed positive associations between the child’s age at first use of a mobile device and the child’s and parent’s age at the time of survey. Negative associations were observed between the health-related quality of life score and sleep problems score. The age of the children at first use of a mobile device differed by their sex, history of developmental concerns assessed at health checkups, possession of personal mobile devices, and mobile device restriction on weekdays and holidays. When the children were stratified by school type, their age at first use of a mobile device was associated with the child’s and parent’s age at the time of survey, their sex, their history of developmental concerns assessed at health checkups, their health-related quality of life, their sleep problems, and their internet addiction, among at least one school type (Table 4).

**Table 3:** Number (%) of child behavioral problems based on the TDS and subscales according to SDQ.

	**Categories**	**Overall**	**School type**			**p**

			**Elementary school**	**Junior high school**	**Senior high school**	
			
		**Number (%)**	**Number (%)**	**Number (%)**	**Number (%)**	
TDS	Normal	2586 (85.6)	1205 (84.1)	972 (86.7)	409 (87.6)	0.072
	Borderline/High	435 (14.4)	228 (15.9)	149 (13.3)	58 (12.4)	

Conduct problems	Normal	2727 (90.3)	1260 (87.9)	1032 (92.1)	435 (93.1)	<0.001
	Borderline/High	294 (9.7)	173 (12.1)	89 (7.9)	32 (6.9)	

Hyperactivity/inattention	Normal	2727 (90.3)	1257 (87.7)	1036 (92.4)	434 (92.9)	<0.001
	Borderline/High	294 (9.7)	176 (12.3)	85 (7.6)	33 (7.1)	

Emotional problems	Normal	2598 (86.0)	1228 (85.7)	964 (86.0)	406 (86.9)	0.798
	Borderline/High	423 (14.0)	205 (14.3)	157 (14.0)	61 (13.1)	

Peer problems	Normal	2594 (85.9)	1255 (87.6)	949 (84.7)	390 (83.5)	0.031
	Borderline/High	427 (14.1)	178 (12.4)	172 (15.3)	77 (16.5)	

Prosocial behavior	Normal	2006 (66.6)	1011 (70.7)	715 (64.0)	280 (60.3)	<0.001
	Borderline/High	1005 (33.4)	418 (29.3)	403 (36.0)	184 (39.7)	

TDS, Total difficulties score. SDQ, Strength and Difficulties Questionnaire. P value by χ^2^ test, which was used to compare the data among elementary, junior high, and senior high school children.

**Table 4:** Associations between child’s age at first use of a mobile device and characteristics of participants.

	**Categories**	**All children**		**School type**					

				**Elementary school children**		**Junior high school children**		**High school children**	
			
		**Median (IQR)**	**r**	**Median (IQR)**	**r**	**Median (IQR)**	**r**	**Median (IQR)**	**r**
Child									
Age at time of survey			0.466**		0.272**		0.131**		−0.031

Sex	Boy	6.0 (5.0, 10.0)**		5.0 (3.0, 7.0)**		8.0 (5.0, 10.0)**		10.0 (6.0, 12.0)**	
	Girl	7.0 (5.0, 10.0)		6.0 (4.0, 8.0)		10.0 (6.0, 12.0)		10.0 (7.0, 12.0)	

Siblings	No	7.0 (5.0, 10.0)		6.0 (4.0, 8.0)		8.0 (6.0, 11.0)		10.5 (6.0, 12.3)	
	Yes	7.0 (5.0, 10.0)		6.0 (4.0, 8.0)		9.0 (6.0, 11.0)		10.0 (7.0, 12.0)	

History of developmental concerns	No	7.0 (5.0, 10.0)**		6.0 (4.0, 8.0)		9.0 (6.0, 11.0)*		10.0 (7.0, 12.0)	
	Yes	6.0 (5.0, 9.0)		5.5 (3.0, 7.0)		7.0 (6.0, 10.0)		10.0 (6.0, 12.0)	

Personal mobile device use and restriction									
Having personal mobile devices	No	6.0 (4.0, 8.0)**		6.0 (4.0, 7.0)		8.0 (6.0, 10.0)		15.0	
	Yes	7.0 (5.0, 10.0)		6.0 (3.0, 8.0)		9.0 (6.0, 11.0)		10.0 (7.0, 12.0)	

Restricted use of mobile devices on weekdays	No	8.0 (5.0, 10.0)**		6.0 (4.0, 8.0)		9.0 (6.0, 11.0)		10.0 (6.0, 12.0)	
	Yes	7.0 (5.0, 9.0)		6.0 (4.0, 8.0)		9.0 (6.0, 11.0)		10.0 (7.0, 13.0)	

Restricted use of mobile devices on holidays	No	7.5 (5.0, 10.0)**		6.0 (4.0, 8.0)		9.0 (6.0, 11.0)		10.0 (6.0, 12.0)	
	Yes	7.0 (5.0, 9.0)		6.0 (4.0, 8.0)		9.0 (6.0, 11.0)		10.0 (7.3, 13.0)	
Child health quality									
Health-related quality of life			−0.097**		0.013		−0.072*		−0.080
Sleep problems			−0.118**		−0.054*		−0.048		−0.113*
Internet addiction			0.002		−0.118**		−0.072*		−0.024

Mother									
Age at time of survey			0.251**		0.157**		0.061*		0.030

Educational level	≤9	7.0 (5.0, 10.0)		5.0 (4.0, 8.0)		8.0 (5.0, 10.0)		10.0 (6.0, 12.0)	
	10–12	7.0 (5.0, 10.0)		6.0 (4.0, 7.0)		8.0 (6.0, 11.0)		10.0 (6.0, 12.0)	
	13–15	7.0 (5.0, 10.0)		6.0 (4.0, 8.0)		9.0 (6.0, 11.0)		10.0 (7.0, 12.0)	
	>16	7.0 (5.0, 10.0)		6.0 (4.0, 8.0)		9.0 (6.0, 11.0)		10.5 (7.8, 14.0)	
Father									
Age at time of survey			0.251**		0.144**		0.112**		0.082

Educational level	≤9	7.0 (5.0, 10.0)		5.0 (3.8, 7.0)		10.0 (6.3, 12.0)		10.0 (7.0, 12.0)	
	10–12	7.0 (5.0, 10.0)		6.0 (3.0, 7.0)		9.0 (6.0, 11.0)		10.0 (6.0, 12.0)	
	13–15	7.0 (5.0, 10.0)		6.0 (4.0, 8.0)		8.0 (6.0, 10.0)		10.0 (6.0, 12.0)	
	>16	7.0 (5.0, 10.0)		6.0 (4.0, 8.0)		9.0 (6.0, 11.0)		10.0 (7.0, 12.0)	

Annual household income (million Japanese Yen)	<3.0	7.0 (5.0, 10.0)		6.0 (4.0, 8.0)		9.0 (6.0, 11.0)		10.0 (6.0, 12.0)	
	3.0–4.9	7.0 (5.0, 10.0)		6.0 (4.0, 7.0)		9.0 (6.0, 11.0)		10.0 (6.0, 12.0)	
	5.0–7.9	7.0 (5.0, 10.0)		6.0 (4.0, 8.0)		9.0 (6.0, 11.0)		10.0 (7.0, 13.0)	
	≥8	7.0 (5.0, 10.0)		6.0 (3.8, 8.0)		9.0 (6.0, 11.0)		10.0 (6.5, 12.0)	

IQR, interquartile range is the 75th and 25th percentile. r, Spearman’s rank correlation coefficient.

*P < 0.05, **P < 0.01 by one-way ANOVA, χ^2^ test, and Kruskal-Wallis test, which were used to compare data among all children, and among those stratified by school type

The associations between child behavioral problems (TDS) and the characteristics of participants among all children and among those stratified by school type are shown in Supplemental Tables 2 and 3. Among all children, the prevalence of child behavioral problems differed by the child’s and mother’s age at the time of survey, their sex, presence of siblings, their history of developmental concerns assessed by medical checkup, mobile device restrictions on weekdays and holidays, their health-related quality of life, their sleep problems, their internet addiction, and their maternal educational levels (Supplemental Tables 2 and 3). When stratified by school type, the prevalence of the children’s behavioral problems differed by the children’s age at the time of survey, their sex, presence of siblings, their history of developmental concerns assessed at health checkups, mobile device restrictions on weekdays and holidays, their health-related quality of life, their sleep problems, and their internet addiction, among at least one school type (Supplemental Tables 2 and 3).

**Fig. 2:**
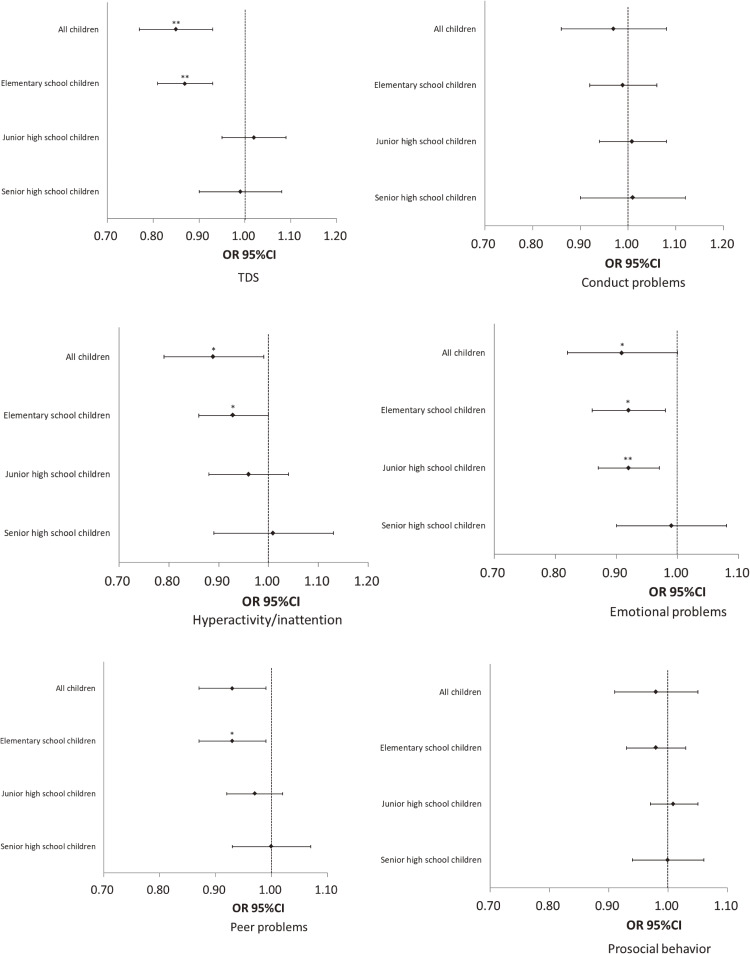
Behavioral problems in children and child age at first use of a mobile device OR: odds ratio. CI; confidence interval. TDS; total each difficulties score. The OR (95% CI) for children of borderline/high group, who was compared to children of normal group based on total each TDS and the five subscales—conduct problems, hyperactivity, emotional symptoms, peer problems, and prosocial behavior were calculated by the logistic regression analysis, which was adjusted for child age at time of survey, sex, history of developmental concerns, health-related quality of life, sleep problems, internet addiction, school type, and interaction between exposure and school type among all children. The logistic regression analysis among children stratified by school type was adjusted for the same variables excluding school type and interaction between exposure and school type. *P < 0.05, **P < 0.01

According to the logistic regression analysis among all children, compared to the normal group, the adjusted odds ratios of the borderline/high group significantly decreased with increasing child age at first use of a mobile device (95% CI = 0.85 [0.77, 0.93]); by contrast, it significantly increased with increasing duration for use of a mobile device (95% CI = 1.20 [1.08, 1.33]) (Table 5 and Fig. 2). In the logistic regression analysis stratified by school type, compared to the normal group, the adjusted odds ratios of child behavioral problems for the borderline/high group significantly decreased with increasing child’s age at first use of a mobile device (95% CI = 0.87 [0.81, 0.93]) and significantly increased with increasing duration for use of a mobile device (95% CI = 1.15 [1.08, 1.23]) among elementary school children. However, the adjusted odds ratios for the child behavioral problems were not significantly associated with the child’s age and duration of mobile device use among junior and senior high school children (Table 5 and Fig. 2). The effects of the interaction between exposure (age at first use of a mobile device and the duration) and the school type were statistically significant (Table 5, and Supplemental Table 4 and 8).

**Table 5:** OR for child behavioral problems (TDS) according to child’s mobile device usage.

**Exposure**	**All children**		**Elementary school**	**Junior high school**	**Senior high school**
			
	**OR (95% CI)a**	**P for interaction**	**OR (95% CI)b**	**OR (95% CI)b**	**OR (95% CI)b**
Age at first use of a mobile device	0.85 (0.77, 0.93)**	0.009	0.87 (0.81, 0.93)**	1.02 (0.96, 1.09)	0.99 (0.91, 1.08)
Duration for use of a mobile device	1.20 (1.08, 1.33)**	0.005	1.15 (1.08, 1.23)**	0.98 (0.92, 1.04)	1.01 (0.93, 1.10)
Age at first having personal mobile devices	0.94 (0.79, 1.11)	0.776	0.89 (0.77, 1.02)	0.95 (0.85, 1.05)	0.90 (0.80, 1.02)
Duration for having personal mobile devices	1.10 (0.90, 1.34)	0.988	1.13 (0.98, 1.30)	1.06 (0.95, 1.17)	1.11 (0.98, 1.25)

TDS; total difficulties score.

a (ALL); The logistic regression analysis models were adjusted for child age at time of survey, sex, history of developmental concerns, health-related quality of life, sleep problems, internet addiction, school type, and interaction between exposure and school type.

b (Stratified by school type); The logistic regression analysis models were adjusted for child age at time of survey, sex, history of developmental concerns, health-related quality of life, sleep problems, and internet addiction.

*P < 0.05, **P < 0.01

P for interaction; each exposure (child’s age at their first use of a mobile device and duration of use) and school type.

The adjusted odds ratios of the five subscales—conduct problems, hyperactivity, emotional symptoms, peer problems, and prosocial behavior—according to the logistic regression analysis results for all children and those stratified by the school type are shown in Table 6 and Fig. 2 (for the borderline/high and normal groups). Among all the children and among elementary school children only, the adjusted odds ratios for hyperactivity and peer problems significantly decreased with increasing child’s age at first use of a mobile device and decreasing child’s duration for the use of a mobile device. Among all the children and among those in elementary and junior high school, the adjusted odds ratios for emotional symptoms significantly decreased with increasing age at first use of a mobile device and decreasing duration for use of a mobile device. Among all the children in senior high school, the adjusted odds ratios for conduct problems significantly decreased with increasing age at first use of personal mobile devices and decreasing duration for having personal mobile devices (Table 6). In the supplemental logistic regression analysis stratified by the children’s sex, boys who were younger at their first use of a mobile device and used such devices for longer durations were found to be hyperactive, have emotional instabilities, and experience peer problems in elementary school but displayed opposite behaviors in senior high school. Girls who were younger at their first use of mobile devices and used such devices for longer durations were found to have emotional instabilities in junior high school but displayed opposite behaviors in senior high school (Supplemental Tables 4–7).

**Table 6:** OR of subscale of SDQ according to child’s mobile device usage.

**Exposure**	**All children**		**Elementary school**	**Junior high school**	**Senior high school**
			
	**OR (95% CI)a**	**P for interaction**	**OR (95% CI)b**	**OR (95% CI)b**	**OR (95% CI)b**
	Conduct problems				
Age at first use of a mobile device	0.97 (0.87, 1.08)	0.543	0.99 (0.93, 1.06)	1.01 (0.94, 1.08)	1.01 (0.91, 1.12)
Duration of mobile device use	1.02 (0.91, 1.14)	0.741	1.01 (0.94, 1.08)	0.99 (0.93, 1.07)	0.99 (0.89, 1.10)
Age at first having personal mobile devices	0.95 (0.78, 1.15)	0.696	0.91 (0.78, 1.06)	0.95 (0.85, 1.08)	0.87 (0.76, 0.99)*
Duration of having personal mobile devices	1.07 (0.85, 1.33)	0.831	1.11 (0.95, 1.30)	1.05 (0.93, 1.19)	1.15 (1.01, 1.32)*

	Hyperactivity/inattention				
Age at first use of a mobile device	0.89 (0.79, 0.99)*	0.173	0.93 (0.87, 1.00)*	0.96 (0.89, 1.04)	1.01 (0.90, 1.13)
Duration of mobile device use	1.12 (0.99, 1.25)	0.264	1.08 (1.00, 1.15)*	1.04 (0.96, 1.12)	0.99 (0.88, 1.11)
Age at first having personal mobile devices	0.91 (0.74, 1.12)	0.829	0.94 (0.80, 1.11)	0.91 (0.80, 1.04)	0.95 (0.81, 1.11)
Duration of having personal mobile devices	1.09 (0.86, 1.39)	0.875	1.06 (0.90, 1.25)	1.09 (0.96, 1.24)	1.05 (0.90, 1.23)

	Emotional problems				
Age at first use of a mobile device	0.91 (0.83, 1.00)*	0.541	0.92 (0.87, 0.98)*	0.92 (0.87, 0.97)**	0.99 (0.91, 1.08)
Duration of mobile device use	1.15 (1.04, 1.26)**	0.129	1.08 (1.02, 1.15)*	1.09 (1.03, 1.15)**	1.01 (0.93, 1.10)
Age at first having personal mobile devices	1.01 (0.85, 1.19)	0.652	0.97 (0.84, 1.11)	0.98 (0.88, 1.08)	0.97 (0.86, 1.10)
Duration of having personal mobile devices	1.03 (0.85, 1.26)	0.996	1.03 (0.90, 1.18)	1.03 (0.92, 1.14)	1.03 (0.91, 1.16)

	Peer problems				
Age at first use of a mobile device	0.93 (0.85, 1.02)	0.490	0.93 (0.87, 0.99)*	0.97 (0.92, 1.02)	1.00 (0.92, 1.07)
Duration of mobile device use	1.09 (0.99, 1.20)	0.350	1.07 (1.01, 1.15)*	1.03 (0.98, 1.09)	1.00 (0.93, 1.08)
Age at first having personal mobile devices	0.90 (0.77, 1.06)	0.191	0.93 (0.81, 1.07)	0.96 (0.88, 1.06)	1.13 (0.99, 1.28)
Duration of having personal mobile devices	1.16 (0.96, 1.40)	0.130	1.07 (0.93, 1.23)	1.04 (0.94, 1.14)	0.89 (0.78, 1.01)

	Prosocial behavior				
Age at first use of a mobile device	0.98 (0.91, 1.05)	0.513	0.98 (0.94, 1.03)	1.01 (0.97, 1.05)	1.00 (0.95, 1.06)
Duration of mobile device use	1.01 (0.95, 1.09)	0.690	1.02 (0.97, 1.07)	0.99 (0.95, 1.03)	1.00 (0.95, 1.06)
Age at first having personal mobile devices	1.05 (0.93, 1.19)	0.606	1.05 (0.95, 1.17)	1.04 (0.96, 1.12)	0.99 (0.91, 1.07)
Duration of having personal mobile devices	0.90 (0.77, 1.04)	0.235	0.95 (0.85, 1.06)	0.96 (0.89, 1.04)	1.01 (0.93, 1.10)

SDQ; The strengths and difficulties questionnaire.

a (ALL); The logistic regression analysis models were adjusted for child age at time of survey, sex, history of developmental concerns, health-related quality of life, sleep problems, internet addiction, school type, and interaction between exposure and school type.

b (Stratified by school type); The logistic regression analysis models were adjusted for child age at time of survey, sex, history of developmental concerns, health-related quality of life, sleep problems, and internet addiction.

P for interaction; each exposure (child’s age at their first use of a mobile device and duration of use) and school type.

*P < 0.05, **P < 0.01

## 4 Discussion

In this cross-sectional study, children who were younger at their first use of a mobile device and used such devices for longer durations represented more problematic behaviors according to the SDQ. Moreover, when stratified by school type, the above associations remained statistically significant for elementary school children, but not for junior high school and older children (Table 5). Our results suggest that elementary school children are more sensitive to mobile device usage than junior high and senior high school children because they are in the early stages of socialization and their behavioral and mental development is rapidly growing. Four exposure factors were determined in this study: the child’s age at first use of a mobile device and the duration of use, and the child’s age at their first owning of a personal mobile device and the duration of owning. Given the rapid spread of mobile devices in recent years, elementary school children have started using such devices earlier than high school children did. By contrast, elementary school children used mobile devices for shorter periods than high school children. However, elementary school children represented more problematic behaviors, suggesting that starting to use mobile devices at an early age causes negative effects on developmental immature neural behaviors. Through a cross-sectional and longitudinal survey, the Danish National Birth Cohort has reported negative prenatal and postnatal effects of cell phone use on emotional and behavioral difficulties in children aged 7 and 11 [4, 5, 27]; our study corroborated the results of this study, showing that early exposure to mobile devices can cause developmental impacts on school-aged children.

Using SDQ subscales, our study assessed problematic behaviors among school children aged 7–17 years. Elementary school children who were younger at their first use of a mobile device and used these devices for longer durations had increased hyperactivity–inattention and displayed peer and emotional problematic behaviors. Moreover, junior high school children who were younger at their first use of a mobile device and used such devices for longer durations represented more emotional problematic behaviors. One possible reason for these differences in the relationships with the school type is that the contents of mobile device use may differ by the school type. Different effects with several content types were evaluated as screen time exposure, which has been noted as a risk factor for sensory development impacts, emotional and behavioral problems, sleep disturbances, and internet addiction among school-aged children [6, 9]. A Swedish study targeting teenagers described that using mobile devices for social networking services is associated with increased communication skills as a positive effect; however, it is also associated with increased anxiety as a negative effect [10]. The results of our study corroborated those of the Swedish study, which stated that early exposure to mobile devices could exacerbate emotional instabilities in teenagers. While the duration of personal mobile device usage did not differ by the children’s school type (Table 2), senior high school children who were younger and had a personal mobile device for a longer period represented more conducted problematic behaviors. This suggests a correlation between having a mobile device and exhibiting oppositional and defiant behaviors among high-school teenagers. When stratified by sex, only boys exhibited an association between mobile device usage and hyperactivity and peer problems. Moreover, associations between mobile device usage and emotional problems were observed in elementary school boys and junior high school girls (Supplemental Tables 4–7). The above results may be related to differences in developmental properties and timing based on the children’s sex [16, 19]. The adverse effects of having personal mobile devices are inconclusive in this cross-sectional analysis, and a longitudinal evaluation throughout adolescence is needed in future studies.

Parental supervision of their children’s use of mobile devices is recommended. However, the results of this study remained unchanged even after adjusting the parental usage restrictions for weekdays and holidays (Supplemental Tables 8 and 9); taken together, our study demonstrates the importance of delaying the use of mobile devices rather than imposing parental restrictions. In our study, 15.3% of the children aged ≤3 years were found to have used mobile devices according to their parents, which is lower than the rate of 50.2% for children who used the internet according to a 2019 Japanese survey [18]. This difference may be attributed to this study targeting a wide birth-year period of 10 years and excluding the use of internet with a wired connection (TV or personal computer). In our study, 69.7% of the elementary school-aged children had personal mobile devices, higher than the rates of 49.5% and 41.0% for smartphones and tablets, respectively, in 2019 of Japanese’s survey [18]. This difference may be attributed to the fact that this study included mobile game devices and other devices as well. In addition, during the survey period in 2020, the penetration of mobile devices among the school-aged children in Hokkaido prefecture increased because the administration started to provide mobile devices to a part of school children for online classes.

### 4.1 Strengths

This study was conducted from 2020 to 2021 and allowed us to examine recent associations between mobile device usage and behavioral problems in children aged 7–17 years. This study used four exposure factors, including the age at their first use of a mobile device and the duration of usage before or after owing a personal mobile device. This facilitated the evaluation of both early exposure to and having mobile devices. Previous studies have reported that the early use of mobile devices disrupts healthy activities and increases the risk of sleep problems and internet addiction and lowers the health-related quality of life [2, 3, 31]. Children with developmental disorders often exhibit symptoms such as insomnia and anxiety [1]. These factors may be mutually related with mobile device usage and behavioral problems in children. Our results were obtained after adjusting for mutually related potential confounders, including sleep problems, internet addiction, health-related quality of life, and developmental concerns.

### 4.2 Limitations

This cross-sectional study could not establish a clear causal direction. In fact, a previous study reported that children with developmental disorders are inclined toward heavy use of mobile devices owing to their weak self-regulation and the presence of restricted interests and repetitive behaviors [14]. Also, parents of inattentive and hyperactive children are more likely to use mobile devices when calming down their children [20]. Furthermore, in this study, the parents retrospectively provided the age at which their children first used a mobile device and the duration of use; hence, recall bias may be possible. The differing results by school type may be associated with differences in the sample size, the rate of having personal mobile devices, and the varying content used among school types.

## 5 Conclusions

Our findings suggest that elementary school children are more sensitive to mobile device usage than older children. Children who are younger at their first use of a mobile device and use such devices for longer durations may be prone to emotional instabilities as teenagers. Children who are younger and have had a personal mobile device for longer may show oppositional behaviors as teenagers. However, longitudinal follow-up studies are needed to clarify whether these problems disappear with age.

## Supplementary information

**Additional file 1:** **Supplemental Table 1.** Distribution of child’s age at first use of a mobile device. **Supplemental Table 2.** Associations between child behavioral problems (TDS) and characteristics of participants (continuous variable). **Supplemental Table 3.** Associations between child behavioral problems (TDS) and characteristics of participants (categorical variable). **Supplemental Table 4.** OR for child behavioral problems (TDS) according to the child’s mobile device usage (boys). **Supplemental Table 5.** OR for subscales of SDQ according to children’s mobile device usage (boys). **Supplemental Table 6.** OR for child behavioral problems (TDS) according to child’s mobile device usage (girls). **Supplemental Table 7.** OR of subscale of SDQ according to child’s mobile device usage (girls). **Supplemental Table 8.** OR for child behavioral problems (TDS) according to child’s mobile device usage (Additional adjustment). **Supplemental Table 9.** OR of subscale of SDQ according to child’s mobile device usage (Additional adjustment).

## Declaration

### Availability of data and material

The datasets generated and/or analyzed during the current study are not publicly available because the study involves human participants with a nondisclosure provision of individual data stated in the written informed consent in order to prevent compromise of study participants’ privacy but are available from the corresponding author upon reasonable request.
