# Supplementary material for: Cross-sectional associations between early mobile device usage and problematic behaviors among school-aged children in the Hokkaido Study on Environment and Children’s Health
Source: Environ Health Prev Med. 2023 Apr 12;28:22. doi: 10.1265/ehpm.22-00245 (PMC10106332; doi:10.1265/ehpm.22-00245)
Supplement: Supplementary file 1 — Additional file 1: Supplemental Table 1. Distribution of child’s age at first use of a mobile device. Supplemental Table 2. Associations between child behavioral problems (TDS) and characteristics of participants (continuous variable). Supplemental Table 3. Associations between child behavioral problems (TDS) and characteristics of participants (categorical variable). Supplemental Table 4. OR for child behavioral problems (TDS) according to the child’s mobile device usage (boys). Supplemental Table 5. OR for subscales of SDQ according to children’s mobile device usage (boys). Supplemental Table 6. OR for child behavioral problems (TDS) according to child’s mobile device usage (girls). Supplemental Table 7. OR of subscale of SDQ according to child’s mobile device usage (girls). Supplemental Table 8. OR for child behavioral problems (TDS) according to child’s mobile device usage (Additional adjustment). Supplemental Table 9. OR of subscale of SDQ according to child’s mobile device usage (Additional adjustment). [file ehpm-28-022-s001.docx]

Supplemental Table 1. Distribution of child’s age at first use of a mobile device.

|  | All children |  | School type |  |  |  |  |  |
| --- | --- | --- | --- | --- | --- | --- | --- | --- |
|  |  |  | Elementary school |  | Junior high school |  | Senior high school |  |
| Child age (year) | Number (%) |  | Number (%) |  | Number (%) |  | Number (%) | p |
| 0 | 14 (0.5) |  | 12 (0.8) |  | 2 (0.2) |  | 0 (0.0) | < 0.001 |
| 1 | 75 (2.5) |  | 64 (4.5) |  | 9 (0.8) |  | 2 (0.4) |  |
| 2 | 146 (4.8) |  | 115 (8.0) |  | 25 (2.2) |  | 6 (1.3) |  |
| 3 | 226 (7.5) |  | 155 (10.8) |  | 52 (4.6) |  | 19 (4.1) |  |
| 4 | 176 (5.8) |  | 122 (8.5) |  | 43 (3.8) |  | 11 (2.4) |  |
| 5 | 334 (11.1) |  | 197 (13.7) |  | 104 (9.3) |  | 33 (7.1) |  |
| 6 | 376 (12.4) |  | 194 (13.5) |  | 142 (12.7) |  | 40 (8.6) |  |
| 7 | 355 (11.8) |  | 207 (14.4) |  | 110 (9.8) |  | 38 (8.1) |  |
| 8 | 199 (6.6) |  | 117 (8.2) |  | 60 (5.4) |  | 22 (4.7) |  |
| 9 | 172 (5.7) |  | 103 (7.2) |  | 51 (4.5) |  | 18 (3.9) |  |
| 10 | 421 (13.9) |  | 114 (8.0) |  | 221 (19.7) |  | 86 (18.4) |  |
| 11 | 124 (4.1) |  | 27 (1.9) |  | 75 (6.7) |  | 22 (4.7) |  |
| 12 | 210 (7.0) |  | 6 (0.4) |  | 139 (12.4) |  | 65 (13.9) |  |
| 13 | 107 (3.5) |  | 0 (0.0) |  | 66 (5.9) |  | 41 (8.8) |  |
| 14 | 43 (1.4) |  | 0 (0.0) |  | 20 (1.8) |  | 23 (4.9) |  |
| 15 | 37 (1.2) |  | 0 (0.0) |  | 2 (0.2) |  | 35 (7.5) |  |
| 16 | 6 (0.2) |  | 0 (0.0) |  | 0 (0.0) |  | 6 (1.3) |  |

p value by χ^2^ test, which was used to compare data among elementary, junior high, and senior high school children.

Supplemental Table 2. Associations between child behavioral problems (TDS) and characteristics of participants (continuous variable)

|  | All children |  |  |  | School type | | |  |  | | |  |  |  |  |
| --- | --- | --- | --- | --- | --- | --- | --- | --- | --- | --- | --- | --- | --- | --- | --- |
|  |  |  |  |  | Elementary school children | | |  | Junior high school children | | |  | Senior high school children | | |
|  | Normal |  | Borderline/High |  | Normal |  | Borderline/ High |  | Normal |  | Borderline/ High |  | Normal |  | Borderline/ High |
|  | Mean ± SD or  Median (IQR) |  | Mean ± SD or  Median (IQR) |  | Mean ± SD or  Median (IQR) |  | Mean ± SD or  Median (IQR) |  | Mean ± SD or  Median (IQR) |  | Mean ± SD or  Median (IQR) |  | Mean ± SD or  Median (IQR) |  | Mean ± SD or  Median (IQR) |
| Child |  |  |  |  |  |  |  |  |  |  |  |  |  |  |  |
| Age at time of survey | 12.4 ± 2.4 |  | 12.1 ± 2.4** |  | 10.2 ± 1.2 |  | 10.1 ± 1.2 |  | 13.7 ± 0.9 |  | 13.6 ± 0.9 |  | 15.8 ± 0.5 |  | 15.8 ± 0.5 |
|  |  |  |  |  |  |  |  |  |  |  |  |  |  |  |  |
| Child health quality |  |  |  |  |  |  |  |  |  |  |  |  |  |  |  |
| Health-related quality of life | 44.0 (39.0, 47.0) |  | 39.0 (34.0, 44.0)** | | 45.0 (40.0, 48.0) |  | 40.0 (36.0, 45.0)** |  | 43.0 (38.0, 47.0) | | 38.0 (33.0, 43.5)** | | 41.0 (37.0, 46.0) |  | 36.0 (30.8, 41.0)** |
| Sleep problems | 23.0 (22.0, 25.0) |  | 26.0 (24.0, 28.0)** | | 24.0 (22.0, 26.0) |  | 26.0 (24.0, 29.0)** |  | 23.0 (21.0, 25.0) | | 26.0 (23.0, 28.0)** | | 23.0 (21.0, 25.0) |  | 26.0 (23.0, 28.0)** |
| Internet addiction | 20.0 (16.0, 25.0) |  | 24.0 (20.0, 30.0)** | | 19.0 (14.5, 24.0) |  | 23.0 (19.0, 28.0)** |  | 21.0 (17.0, 26.0) | | 25.0 (21.0, 30.0)** | | 22.0 (18.0, 27.0) |  | 26.0 (20.0, 31.3)** |
|  |  |  |  |  |  |  |  |  |  |  |  |  |  |  |  |
| Mother |  |  |  |  |  |  |  |  |  |  |  |  |  |  |  |
| Age at time of survey | 44.1 ± 5.1 |  | 43.6 ± 5.2* |  | 42.2 ± 4.9 |  | 41.6 ± 4.8 |  | 45.3 ± 4.5 |  | 45.2 ± 5.0 |  | 47.2 ± 4.3 |  | 47.4 ± 3.7 |
| Father |  |  |  |  |  |  |  |  |  |  |  |  |  |  |  |
| Age at time of survey | 45.7 ± 5.9 |  | 45.3 ± 5.6 |  | 43.7 ± 5.7 |  | 43.4 ± 5.6 |  | 46.9 ± 5.5 |  | 46.9 ± 5.1 |  | 48.9 ± 5.3 |  | 48.3 ± 4.3 |

SD, standard deviation. TDS; total difficulties score. IQR, interquartile range is the 75th and 25th percentile.

*P < 0.05, **P < 0.01 by one-way ANOVA, χ^2^ test, and Kruskal-Wallis test, which were used to compare data among all children and among those stratified by school type

Supplemental Table 3. Associations between child behavioral problems (TDS) and characteristics of participants (categorical variable)

|  |  | All children |  |  |  | School type | | |  |  | | |  |  | | |
| --- | --- | --- | --- | --- | --- | --- | --- | --- | --- | --- | --- | --- | --- | --- | --- | --- |
|  |  |  |  |  |  | Elementary school children | | |  | Junior high school children | | |  | Senior school children | | |
|  |  | Normal |  | Borderline/High |  | Normal |  | Borderline/High |  | Normal |  | Borderline/High |  | Normal |  | Borderline/High |
|  |  | Number (%) |  | Number (%) |  | Number (%) |  | Number (%) |  | Number (%) |  | Number (%) |  | Number (%) |  | Number (%) |
| Child |  |  |  |  |  |  |  |  |  |  |  |  |  |  |  |  |
| Sex | Boy | 1252 (83.5) |  | 247 (16.5)** |  | 578 (80.2) |  | 143 (19.8)** |  | 473 (86.0) |  | 77 (14.0)** |  | 201 (88.2) |  | 27 (11.8)** |
|  | Girl | 1334 (87.6) |  | 188 (12.4) |  | 627 (88.1) |  | 85 (11.9) |  | 499 (87.4) |  | 72 (12.6) |  | 208 (87.0) |  | 31 (13.0) |
|  |  |  |  |  |  |  |  |  |  |  |  |  |  |  |  |  |
| Siblings | No | 322 (80.3) |  | 79 (19.7)** |  | 147 (76.6) |  | 45 (23.4)** |  | 127 (84.1) |  | 24 (15.9)** |  | 48 (82.8) |  | 10 (17.2)** |
|  | Yes | 1819 (86.6) |  | 281 (13.4) |  | 792 (85.5) |  | 134 (14.5) |  | 723 (87.1) |  | 107 (12.9) |  | 304 (88.4) |  | 40 (11.6) |
|  |  |  |  |  |  |  |  |  |  |  |  |  |  |  |  |  |
| History of developmental concerns | No | 2402 (88.1) |  | 324 (11.9)** |  | 1108 (87.2) | | 163 (12.8)** |  | 912 (88.7) |  | 116 (11.3)** |  | 382 (89.5) |  | 45 (10.5)** |
|  | Yes | 184 (62.4) |  | 111 (37.6) |  | 97 (59.9) |  | 65 (40.1) |  | 60 (64.5) |  | 33 (35.5) |  | 27 (67.5) |  | 13 (32.5) |
|  |  |  |  |  |  |  |  |  |  |  |  |  |  |  |  |  |
| Personal mobile device use and restriction |  |  |  |  |  |  |  |  |  |  |  |  |  |  |  |  |
| Having personal mobile devices | No | 479 (84.9) |  | 85 (15.1) |  | 369 (85.0) |  | 65 (15.0) |  | 109 (84.5) |  | 20 (15.5) |  | 1 (100.0) |  | 0 (0.0) |
|  | Yes | 2107 (85.8) |  | 350 (14.2) |  | 836 (83.7) |  | 163 (16.3) |  | 863 (87.0) |  | 129 (13.0) |  | 408 (87.6) |  | 58 (12.4) |
|  |  |  |  |  |  |  |  |  |  |  |  |  |  |  |  |  |
| Restricted use of mobile devices on weekdays | No | 1142 (87.2) |  | 168 (12.8)* |  | 325 (83.5) |  | 64 (16.5)** |  | 478 (88.5) |  | 62 (11.5)** |  | 339 (89.0) |  | 42 (11.0)** |
|  | Yes | 1442 (84.4) |  | 267 (15.6) |  | 878 (84.3) |  | 164 (15.7) |  | 494 (85.0) |  | 87 (15.0) |  | 70 (81.4) |  | 16 (18.6) |
|  |  |  |  |  |  |  |  |  |  |  |  |  |  |  |  |  |
| Restricted use of mobile devices on holidays | No | 1271 (87.3) |  | 185 (12.7)** |  | 400 (84.7) |  | 72 (15.3)** |  | 521 (88.5) |  | 68 (11.5)** |  | 350 (88.6) |  | 45 (11.4)** |
|  | Yes | 1315 (84.0) |  | 250 (16.0) |  | 805 (83.8) |  | 156 (16.2) |  | 451 (84.8) |  | 81 (15.2) |  | 59 (81.9) |  | 13 (18.1) |
| Mother |  |  |  |  |  |  |  |  |  |  |  |  |  |  |  |  |
| Educational level | ≤ 9 | 67 (83.8) |  | 13 (16.3)* |  | 29 (78.4) |  | 8 (21.6)** |  | 27 (84.4) |  | 5 (15.6) |  | 11 (100.0) |  | 0 (0.0)* |
|  | 10–12 | 965 (83.8) |  | 187 (16.2) |  | 441 (79.7) |  | 112 (20.3) |  | 362 (86.0) |  | 59 (14.0) |  | 162 (91.0) |  | 16 (9.0) |
|  | 13–15 | 1205 (87.6) |  | 171 (12.4) |  | 552 (87.6) |  | 78 (12.4) |  | 458 (87.7) |  | 64 (12.3) |  | 195 (87.1) |  | 29 (12.9) |
|  | > 16 | 349 (84.5) |  | 64 (15.5) |  | 183 (85.9) |  | 30 (14.1) |  | 125 (85.6) |  | 21 (14.4) |  | 41 (75.9) |  | 13 (24.1) |
| Father |  |  |  |  |  |  |  |  |  |  |  |  |  |  |  |  |
| Educational level | ≤ 9 | 139 (83.2) |  | 28 (16.8) |  | 68 (82.9) |  | 14 (17.1) |  | 47 (83.9) |  | 9 (16.1) |  | 24 (82.8) |  | 5 (17.2) |
|  | 10–12 | 928 (84.4) |  | 171 (15.6) |  | 414 (81.5) |  | 94 (18.5) |  | 354 (86.6) |  | 55 (13.4) |  | 160 (87.9) |  | 22 (12.1) |
|  | 13–15 | 672 (85.6) |  | 113 (14.4) |  | 332 (86.2) |  | 53 (13.8) |  | 247 (84.6) |  | 45 (15.4) |  | 93 (86.1) |  | 15 (13.9) |
|  | > 16 | 822 (87.6) |  | 116 (12.4) |  | 386 (85.6) |  | 65 (14.4) |  | 310 (89.6) |  | 36 (10.4) |  | 126 (89.4) |  | 15 (10.6) |
|  |  |  |  |  |  |  |  |  |  |  |  |  |  |  |  |  |
| Annual household income (million Japanese Yen) | < 3.0 | 408 (80.0) |  | 102 (20.0)** |  | 194 (77.0) |  | 58 (23.0)** |  | 157 (80.9) |  | 37 (19.1) |  | 57 (89.1) |  | 7 (10.9) |
|  | 3.0–4.9 | 1,054 (85.7) |  | 176 (14.3) |  | 497 (83.7) |  | 97 (16.3) |  | 390 (87.4) |  | 56 (12.6) |  | 167 (87.9) |  | 23 (12.1) |
|  | 5.0–7.9 | 628 (87.0) |  | 94 (13.0) |  | 301 (86.7) |  | 46 (13.3) |  | 229 (87.1) |  | 34 (12.9) |  | 98 (87.5) |  | 14 (12.5) |
|  | ≥ 8 | 203 (89.8) |  | 23 (10.2) |  | 91 (92.9) |  | 7 (7.1) |  | 76 (87.4) |  | 11 (12.6) |  | 36 (87.8) |  | 5 (12.2) |

TDS; total difficulties score.

*P < 0.05, **P < 0.01 by one-way ANOVA, χ^2^ test, and Kruskal-Wallis test, which were used to compare data among all children and among those stratified by school type

Supplemental Table 4. OR for child behavioral problems (TDS) according to the child’s mobile device usage (boys).

|  |  | Overall |  |  | Elementary school |  | Junior high school |  | Senior high school |
| --- | --- | --- | --- | --- | --- | --- | --- | --- | --- |
| Exposure |  | OR (95% CI)a | P for interaction |  | OR (95% CI)b |  | OR (95% CI)b |  | OR (95% CI)b |
| Age at first use of a mobile device |  | 0.83 (0.73, 0.95)** | 0.021 |  | 0.87 (0.81, 0.93)** |  | 1.03 (0.94, 1.13) |  | 1.03 (0.91, 1.17) |
| Duration of mobile device usage |  | 1.23 (1.07, 1.42)** | 0.013 |  | 1.15 (1.08, 1.23)** |  | 0.97 (0.89, 1.06) |  | 0.97 (0.85, 1.10) |
| Age at first having a personal mobile device |  | 0.90 (0.70, 1.15) | 0.834 |  | 0.89 (0.77, 1.02) |  | 0.89 (0.77, 1.02) |  | 0.87 (0.73, 1.05) |
| Duration of having personal mobile devices |  | 1.18 (0.88, 1.57) | 0.850 |  | 1.13 (0.98, 1.30) |  | 1.13 (0.98, 1.31) |  | 1.14 (0.95, 1.37) |

TDS; total difficulties score.

a (ALL); The logistic regression analysis models were adjusted for the child’s age at the time of survey, history of developmental concerns, health-related quality of life, sleep problems, internet addiction, school type, and the interaction between exposure and school type.

b (Stratified by school type); The logistic regression analysis models were adjusted for the child’s age at the time of survey, history of developmental concerns, health-related quality of life, sleep problems, and internet addiction.

*P < 0.05, **P < 0.01

P for interaction; each exposure (child’s age at their first use of a mobile device and the duration of use) and school type.

Supplemental Table 5. OR for subscales of SDQ according to children’s mobile device usage (boys).

|  |  | Overall |  |  | Elementary school |  | Junior high school |  | Senior high school |
| --- | --- | --- | --- | --- | --- | --- | --- | --- | --- |
| Exposure |  | OR (95% CI)a | P for interaction |  | OR (95% CI)b |  | OR (95% CI)b |  | OR (95% CI)b |
|  |  | Conduct problems |  |  |  |  |  |  |  |
| Age at first use of a mobile device |  | 0.94 (0.82, 1.08) | 0.051 |  | 1.01 (0.93, 1.11) |  | 1.07 (0.97, 1.18) |  | 1.19 (1.01, 1.39)* |
| Duration of mobile device usage |  | 1.06 (0.91, 1.24) | 0.084 |  | 0.99 (0.90, 1.08) |  | 0.93 (0.85, 1.03) |  | 0.84 (0.72, 0.99)* |
| Age at first having a personal mobile device |  | 0.85 (0.65, 1.11) | 0.414 |  | 0.89 (0.71, 1.11) |  | 0.89 (0.76, 1.04) |  | 1.05 (0.82, 1.33) |
| Duration of having a personal mobile device |  | 1.27 (0.93, 1.75) | 0.243 |  | 1.12 (0.90, 1.40) |  | 1.12 (0.96, 1.31) |  | 0.95 (0.75, 1.21) |
|  |  |  |  |  |  |  |  |  |  |
|  |  | Hyperactivity/inattention |  |  |  |  |  |  |  |
| Age at first use of a mobile device |  | 0.87 (0.76, 1.00)* | 0.153 |  | 0.91 (0.84, 0.99)* |  | 0.98 (0.89, 1.08) |  | 1.02 (0.88, 1.18) |
| Duration of mobile device usage |  | 1.16 (1.01, 1.34)* | 0.140 |  | 1.10 (1.01, 1.20)* |  | 1.02 (0.93, 1.12) |  | 0.98 (0.84, 1.13) |
| Age at first having a personal mobile device |  | 0.83 (0.64, 1.06) | 0.317 |  | 0.90 (0.73, 1.10) |  | 0.92 (0.78, 1.08) |  | 1.00 (0.80, 1.25) |
| Duration of having a personal mobile device |  | 1.23 (0.91, 1.67) | 0.351 |  | 1.11 (0.91, 1.36) |  | 1.09 (0.92, 1.29) |  | 1.00 (0.80, 1.25) |
|  |  |  |  |  |  |  |  |  |  |
|  |  | Emotional problems |  |  |  |  |  |  |  |
| Age at first use of a mobile device |  | 0.80 (0.69, 0.93)** | 0.088 |  | 0.85 (0.77, 0.93)** |  | 0.90 (0.81, 1.00)* |  | 0.99 (0.86, 1.15) |
| Duration of mobile device usage |  | 1.28 (1.10, 1.49)** | 0.054 |  | 1.18 (1.07, 1.29)** |  | 1.11 (1.00, 1.23)* |  | 1.01 (0.87, 1.17) |
| Age at first having a personal mobile device |  | 0.93 (0.71, 1.22) | 0.981 |  | 0.91 (0.74, 1.12) |  | 1.02 (0.84, 1.22) |  | 0.86 (0.71, 1.05) |
| Duration of having a personal mobile device |  | 1.05 (0.76, 1.44) | 0.832 |  | 1.10 (0.89, 1.35) |  | 0.99 (0.82, 1.19) |  | 1.16 (0.96, 1.41) |
|  |  |  |  |  |  |  |  |  |  |
|  |  | Peer problems |  |  |  |  |  |  |  |
| Age at first use of a mobile device |  | 0.89 (0.78, 1.02) | 0.459 |  | 0.89 (0.81, 0.98)* |  | 0.97 (0.89, 1.04) |  | 0.95 (0.86, 1.06) |
| Duration of mobile device usage |  | 1.12 (0.98, 1.28) | 0.496 |  | 1.12 (1.02, 1.23)* |  | 1.04 (0.96, 1.12) |  | 1.05 (0.95, 1.16) |
| Age at first having a personal mobile device |  | 0.82 (0.66, 1.03) | 0.109 |  | 0.88 (0.71, 1.08) |  | 0.95 (0.83, 1.09) |  | 1.09 (0.90, 1.30) |
| Duration of having a personal mobile device |  | 1.21 (0.91, 1.59) | 0.239 |  | 1.14 (0.93, 1.40) |  | 1.05 (0.92, 1.21) |  | 0.92 (0.77, 1.11) |
|  |  |  |  |  |  |  |  |  |  |
|  |  | Prosocial behavior |  |  |  |  |  |  |  |
| Age at first use of a mobile device |  | 0.98 (0.89, 1.07) | 0.397 |  | 0.98 (0.92, 1.04) |  | 1.05 (0.99, 1.11) |  | 1.01 (0.93, 1.09) |
| Duration of mobile device usage |  | 1.00 (0.91, 1.10) | 0.731 |  | 1.02 (0.96, 1.09) |  | 0.95 (0.90, 1.01) |  | 0.99 (0.92, 1.07) |
| Age at first having a personal mobile device |  | 1.00 (0.84, 1.18) | 0.943 |  | 1.05 (0.90, 1.22) |  | 0.98 (0.88, 1.09) |  | 1.00 (0.88, 1.13) |
| Duration of having a personal mobile device |  | 0.92 (0.74, 1.13) | 0.414 |  | 0.95 (0.82, 1.11) |  | 1.02 (0.92, 1.14) |  | 1.00 (0.88, 1.13) |

SDQ; The strengths and difficulties questionnaire.

a (ALL); The logistic regression analysis models were adjusted for the child’s age at the time of survey, history of developmental concerns, health-related quality of life, sleep problems, internet addiction, school type, and the interaction between exposure and school type.

b (Stratified by school type); The logistic regression analysis models were adjusted for the child’s age at the time of survey, history of developmental concerns, health-related quality of life, sleep problems, and internet addiction.

P for interaction; each exposure (child’s age at their first use of a mobile device and the duration of use) and school type.

*P < 0.05, **P < 0.01

Supplemental Table 6. OR for child behavioral problems (TDS) according to child’s mobile device usage (girls).

|  |  | All children |  |  | Elementary school |  | Junior high school |  | Senior high school |
| --- | --- | --- | --- | --- | --- | --- | --- | --- | --- |
| Exposure |  | OR (95% CI)a | P for interaction |  | OR (95% CI)b |  | OR (95% CI)b |  | OR (95% CI)b |
| Age at first use of a mobile device |  | 0.88 (0.76, 1.01) | 0.233 |  | 0.86 (0.78, 0.95)** |  | 1.02 (0.94, 1.12) |  | 0.96 (0.85, 1.08) |
| Duration of mobile device usage |  | 1.16 (0.99, 1.35) | 0.186 |  | 1.16 (1.05, 1.28)** |  | 0.98 (0.90, 1.06) |  | 1.05 (0.92, 1.18) |
| Age at first having a personal mobile device |  | 0.97 (0.76, 1.24) | 0.965 |  | 0.91 (0.74, 1.11) |  | 1.07 (0.90, 1.26) |  | 0.94 (0.79, 1.11) |
| Duration of having a personal mobile device |  | 1.04 (0.78, 1.38) | 0.973 |  | 1.10 (0.90, 1.35) |  | 0.94 (0.80, 1.11) |  | 1.07 (0.90, 1.26) |

TDS; total difficulties score.

a (ALL); The logistic regression analysis models were adjusted for the child’s age at the time of survey, history of developmental concerns, health-related quality of life, sleep problems, internet addiction, school type, and the interaction between exposure and school type.

b (Stratified by school type); The logistic regression analysis models were adjusted for the child’s age at the time of survey, history of developmental concerns, health-related quality of life, sleep problems, and internet addiction.

P for interaction; each exposure (child’s age at their first use of a mobile device and the duration of use) and school type.

*P < 0.05, **P < 0.01

Supplemental Table 7. OR of subscale of SDQ according to child’s mobile device usage (girls).

|  |  | All children |  |  | Elementary school |  | Junior high school |  | Senior high school |
| --- | --- | --- | --- | --- | --- | --- | --- | --- | --- |
| Exposure |  | OR (95% CI)a | P for interaction |  | OR (95% CI)b |  | OR (95% CI)b |  | OR (95% CI)b |
|  |  | Conduct problems |  |  |  |  |  |  |  |
| Age at first use of a mobile device |  | 1.02 (0.86, 1.21) | 0.211 |  | 0.95 (0.85, 1.05) |  | 0.93 (0.84, 1.04) |  | 0.83 (0.70, 0.97)* |
| Duration of mobile device usage |  | 0.98 (0.83, 1.16) | 0.178 |  | 1.06 (0.95, 1.18) |  | 1.07 (0.96, 1.19) |  | 1.21 (1.03, 1.43)* |
| Age at first having a personal mobile device |  | 1.06 (0.79, 1.42) | 0.206 |  | 0.88 (0.70, 1.11) |  | 1.08 (0.89, 1.32) |  | 0.73 (0.59, 0.89)** |
| Duration of having a personal mobile device |  | 0.94 (0.68, 1.29) | 0.224 |  | 1.15 (0.92, 1.45) |  | 0.93 (0.76, 1.14) |  | 1.38 (1.13, 1.69)** |
|  |  |  |  |  |  |  |  |  |  |
|  |  | Hyperactivity/inattention |  |  |  |  |  |  |  |
| Age at first use of a mobile device |  | 0.96 (0.78, 1.18) | 0.980 |  | 0.98 (0.86, 1.12) |  | 0.94 (0.82, 1.07) |  | 0.98 (0.82, 1.18) |
| Duration of mobile device usage |  | 1.00 (0.81, 1.24) | 0.723 |  | 1.02 (0.89, 1.16) |  | 1.07 (0.94, 1.22) |  | 1.02 (0.85, 1.22) |
| Age at first having a personal mobile device |  | 1.13 (0.79, 1.63) | 0.334 |  | 1.09 (0.81, 1.48) |  | 0.94 (0.76, 1.16) |  | 0.89 (0.71, 1.13) |
| Duration of having a personal mobile device |  | 0.90 (0.60, 1.35) | 0.454 |  | 0.91 (0.67, 1.24) |  | 1.06 (0.86, 1.31) |  | 1.12 (0.88, 1.42) |
|  |  |  |  |  |  |  |  |  |  |
|  |  | Emotional problems |  |  |  |  |  |  |  |
| Age at first use of a mobile device |  | 1.03 (0.90, 1.17) | 0.257 |  | 0.99 (0.91, 1.08) |  | 0.93 (0.86, 0.99)* |  | 0.98 (0.88, 1.09) |
| Duration of mobile device usage |  | 1.04 (0.92, 1.19) | 0.989 |  | 1.01 (0.92, 1.10) |  | 1.08 (1.01, 1.16)* |  | 1.02 (0.92, 1.14) |
| Age at first having a personal mobile device |  | 1.06 (0.86, 1.32) | 0.561 |  | 1.02 (0.84, 1.23) |  | 0.97 (0.85, 1.11) |  | 1.04 (0.89, 1.22) |
| Duration of having a personal mobile device |  | 1.03 (0.80, 1.33) | 0.795 |  | 0.98 (0.81, 1.18) |  | 1.03 (0.90, 1.18) |  | 0.96 (0.82, 1.12) |
|  |  |  |  |  |  |  |  |  |  |
|  |  | Peer problems |  |  |  |  |  |  |  |
| Age at first use of a mobile device |  | 0.96 (0.84, 1.10) | 0.764 |  | 0.97 (0.88, 1.06) |  | 0.97 (0.90, 1.05) |  | 1.05 (0.94, 1.18) |
| Duration of mobile device usage |  | 1.07 (0.93, 1.23) | 0.478 |  | 1.03 (0.94, 1.13) |  | 1.03 (0.95, 1.11) |  | 0.95 (0.85, 1.07) |
| Age at first having a personal mobile device |  | 0.98 (0.78, 1.23) | 0.706 |  | 0.98 (0.81, 1.19) |  | 0.98 (0.86, 1.12) |  | 1.19 (0.99, 1.44) |
| Duration of having a personal mobile device |  | 1.12 (0.85, 1.46) | 0.314 |  | 1.02 (0.84, 1.24) |  | 1.02 (0.89, 1.16) |  | 0.84 (0.70, 1.01) |
|  |  |  |  |  |  |  |  |  |  |
|  |  | Prosocial behavior |  |  |  |  |  |  |  |
| Age at first use of a mobile device |  | 0.98 (0.88, 1.08) | 0.954 |  | 0.97 (0.90, 1.04) |  | 0.97 (0.92, 1.03) |  | 1.00 (0.92, 1.09) |
| Duration of mobile device usage |  | 1.04 (0.93, 1.15) | 0.762 |  | 1.03 (0.96, 1.11) |  | 1.03 (0.97, 1.09) |  | 1.00 (0.92, 1.08) |
| Age at first having a personal mobile device |  | 1.13 (0.95, 1.35) | 0.380 |  | 1.05 (0.90, 1.22) |  | 1.09 (0.98, 1.22) |  | 0.99 (0.88, 1.12) |
| Duration of having a personal mobile device |  | 0.88 (0.71, 1.08) | 0.414 |  | 0.95 (0.82, 1.11) |  | 0.92 (0.82, 1.02) |  | 1.01 (0.90, 1.13) |

SDQ; The strengths and difficulties questionnaire.

a (ALL); The logistic regression analysis models were adjusted for the child’s age at the time of survey, history of developmental concerns, health-related quality of life, sleep problems, internet addiction, school type, and the interaction between exposure and school type.

b (Stratified by school type); The logistic regression analysis models were adjusted for the child’s age at the time of survey, history of developmental concerns, health-related quality of life, sleep problems, and internet addiction.

P for interaction; each exposure (child’s age at their first use of a mobile device and the duration of use) and school type.

*P < 0.05, **P < 0.01

Supplemental Table 8. OR for child behavioral problems (TDS) according to child’s mobile device usage (Additional adjustment).

|  |  | All children |  |  | Elementary school |  | Junior high school |  | Senior high school |
| --- | --- | --- | --- | --- | --- | --- | --- | --- | --- |
| Exposure |  | OR (95% CI)a | P for interaction |  | OR (95% CI)b |  | OR (95% CI)b |  | OR (95% CI)b |
| Age at first use of a mobile device |  | 0.85 (0.77, 0.93)** | 0.009 |  | 0.87 (0.81, 0.93)** |  | 1.03 (0.97, 1.09) |  | 0.99 (0.91, 1.08) |
| Duration of mobile device usage |  | 1.20 (1.08, 1.33)** | 0.006 |  | 1.15 (1.08, 1.23)** |  | 0.97 (0.92, 1.04) |  | 1.01 (0.92, 1.10) |
| Age at first having a personal mobile device |  | 0.93 (0.78, 1.11) | 0.839 |  | 0.90 (0.78, 1.04) |  | 0.95 (0.86, 1.06) |  | 0.90 (0.80, 1.01) |
| Duration of having a personal mobile device |  | 1.10 (0.89, 1.34) | 0.987 |  | 1.11 (0.96, 1.29) |  | 1.06 (0.95, 1.17) |  | 1.11 (0.99, 1.26) |

TDS; total difficulties score.

a (ALL); The logistic regression analysis models were adjusted for the child’s age at the time of survey, sex, history of developmental concerns, health-related quality of life, sleep problems, internet addiction, school type, the parental usage restrictions for weekdays and holidays, and the interaction between exposure and school type.

b (Stratified by school type); The logistic regression analysis models were adjusted for the child’s age at the time of survey, sex, history of developmental concerns, health-related quality of life, sleep problems, internet addiction, and the parental usage restrictions for weekdays and holidays.

P for interaction; each exposure (child’s age at their first use of a mobile device and the duration of use) and school type.

*P < 0.05, **P < 0.01

Supplemental Table 9. OR of subscale of SDQ according to child’s mobile device usage (Additional adjustment).

|  |  | All children |  |  | Elementary school |  | Junior high school |  | Senior high school |
| --- | --- | --- | --- | --- | --- | --- | --- | --- | --- |
| Exposure |  | OR (95% CI)a | P for interaction |  | OR (95% CI)b |  | OR (95% CI)b |  | OR (95% CI)b |
|  |  | Conduct problems |  |  |  |  |  |  |  |
| Age at first use of a mobile device |  | 0.96 (0.86, 1.07) | 0.445 |  | 0.99 (0.92, 1.06) |  | 1.01 (0.94, 1.09) |  | 1.01 (0.90, 1.12) |
| Duration of mobile device usage |  | 1.02 (0.91, 1.15) | 0.697 |  | 1.02 (0.95, 1.09) |  | 0.99 (0.92, 1.06) |  | 0.99 (0.89, 1.11) |
| Age at first having a personal mobile device |  | 0.96 (0.79, 1.17) | 0.633 |  | 0.92 (0.78, 1.08) |  | 0.96 (0.85, 1.08) |  | 0.87 (0.75, 0.99)* |
| Duration of having a personal mobile device |  | 1.04 (0.83, 1.30) | 0.659 |  | 1.09 (0.93, 1.29) |  | 1.05 (0.93, 1.18) |  | 1.15 (1.01, 1.32)* |
|  |  |  |  |  |  |  |  |  |  |
|  |  | Hyperactivity/inattention |  |  |  |  |  |  |  |
| Age at first use of a mobile device |  | 0.89 (0.79, 0.99)* | 0.157 |  | 0.93 (0.87, 1.00)* |  | 0.97 (0.90, 1.05) |  | 1.01 (0.90, 1.13) |
| Duration of mobile device usage |  | 1.11 (0.99, 1.25) | 0.296 |  | 1.08 (1.00, 1.15)* |  | 1.03 (0.96, 1.11) |  | 0.99 (0.89, 1.11) |
| Age at first having a personal mobile device |  | 0.90 (0.73, 1.11) | 0.728 |  | 0.95 (0.80, 1.12) |  | 0.91 (0.80, 1.04) |  | 0.95 (0.81, 1.12) |
| Duration of having a personal mobile device |  | 1.10 (0.86, 1.40) | 0.853 |  | 1.06 (0.89, 1.25) |  | 1.09 (0.96, 1.24) |  | 1.05 (0.90, 1.23) |
|  |  |  |  |  |  |  |  |  |  |
|  |  | Emotional problems |  |  |  |  |  |  |  |
| Age at first use of a mobile device |  | 0.90 (0.82, 0.99)* | 0.468 |  | 0.92 (0.87, 0.98)** |  | 0.92 (0.87, 0.97)** |  | 0.98 (0.90, 1.07) |
| Duration of mobile device usage |  | 1.15 (1.05, 1.27)** | 0.108 |  | 1.09 (1.02, 1.16)** |  | 1.09 (1.03, 1.15)** |  | 1.02 (0.93, 1.11) |
| Age at first having a personal mobile device |  | 1.01 (0.85, 1.20) | 0.634 |  | 0.98 (0.85, 1.12) |  | 0.98 (0.88, 1.08) |  | 0.97 (0.86, 1.10) |
| Duration of having a personal mobile device |  | 1.02 (0.84, 1.25) | 0.963 |  | 1.02 (0.89, 1.18) |  | 1.03 (0.92, 1.14) |  | 1.03 (0.91, 1.16) |
|  |  |  |  |  |  |  |  |  |  |
|  |  | Peer problems |  |  |  |  |  |  |  |
| Age at first use of a mobile device |  | 0.93 (0.85, 1.02) | 0.513 |  | 0.93 (0.87, 1.00)* |  | 0.97 (0.92, 1.02) |  | 1.00 (0.93, 1.08) |
| Duration of mobile device usage |  | 1.09 (0.99, 1.20) | 0.349 |  | 1.07 (1.00, 1.14)* |  | 1.03 (0.98, 1.09) |  | 1.00 (0.93, 1.08) |
| Age at first having a personal mobile device |  | 0.90 (0.77, 1.06) | 0.187 |  | 0.94 (0.82, 1.08) |  | 0.96 (0.88, 1.06) |  | 1.14 (1.00, 1.30)* |
| Duration of having a personal mobile device |  | 1.16 (0.96, 1.41) | 0.127 |  | 1.06 (0.92, 1.22) |  | 1.04 (0.94, 1.14) |  | 0.88 (0.77, 1.00)* |
|  |  |  |  |  |  |  |  |  |  |
|  |  | Prosocial behavior |  |  |  |  |  |  |  |
| Age at first use of a mobile device |  | 0.98 (0.92, 1.05) | 0.570 |  | 0.98 (0.94, 1.03) |  | 1.01 (0.97, 1.05) |  | 1.00 (0.95, 1.06) |
| Duration of mobile device usage |  | 1.02 (0.95, 1.09) | 0.664 |  | 1.02 (0.97, 1.07) |  | 0.99 (0.95, 1.03) |  | 1.00 (0.95, 1.06) |
| Age at first having a personal mobile device |  | 1.06 (0.94, 1.20) | 0.516 |  | 1.06 (0.95, 1.18) |  | 1.04 (0.96, 1.12) |  | 0.98 (0.90, 1.07) |
| Duration of having a personal mobile device |  | 0.90 (0.78, 1.05) | 0.260 |  | 0.94 (0.85, 1.05) |  | 0.97 (0.89, 1.04) |  | 1.02 (0.93, 1.11) |

SDQ; The strengths and difficulties questionnaire.

a (ALL); The logistic regression analysis models were adjusted for the child’s age at the time of survey, sex, history of developmental concerns, health-related quality of life, sleep problems, internet addiction, school type, the parental usage restrictions for weekdays and holidays, and the interaction between exposure and school type.

b (Stratified by school type); The logistic regression analysis models were adjusted for the child’s age at the time of survey, sex, history of developmental concerns, health-related quality of life, sleep problems, internet addiction, and the parental usage restrictions for weekdays and holidays.

P for interaction; each exposure (child’s age at their first use of a mobile device and the duration of use) and school type.

*P < 0.05, **P < 0.01
